# Learning Urogenital Diseases in Oddity (LUDO)—a gamification-based innovation for learning urogenital diseases in emergency medicine

**DOI:** 10.1186/s12245-023-00567-0

**Published:** 2024-01-09

**Authors:** Asjad Mallick, Shahan Waheed

**Affiliations:** 1 Karachi Medical And Dental College (Abbasi Shaheed Hospital), Karachi, Pakistan; 2 Aga Khan University Hospital, Karachi, Pakistan

**Keywords:** Gamification, Emergency medicine, LUDO, Urogenital diseases, Teamwork

## Abstract

Urogenital emergencies demand immediate attention within the field of emergency medicine, encompassing a range of critical conditions from ectopic pregnancies to kidney stones. Timely diagnosis and treatment are vital to prevent potential mortality and morbidity. However, due to the sensitive nature of these disorders and the cultural taboos surrounding them, accessing prompt medical care can be challenging. To bridge this gap, innovative gamification-based learning techniques, such as the Learning Urogenital Diseases in Oddity (LUDO), have been introduced for emergency medicine residents (Chou, What is gamification? Yukai Chou: Gamification and behavioral design, n.d.; Gamification '13: Proceedings of the first international conference on gameful design, research, and applications, 2013).

LUDO is a timed, gamified exercise that offers residents an interactive and engaging platform to enhance their clinical knowledge related to urogenital disorders. Adapted from the well-known board game, LUDO fosters learning, collaboration, and cooperation among residents. This format is highly customizable and can be utilized by various learning groups.

Participants, emergency medicine residents from different academic years, formed four teams, each distinguished by a unique color. The exercise utilized simple and accessible materials, including a LUDO board, LED stopwatch, laptop, colored hats, and a desk bell. Teams advanced their tokens through the board by correctly answering urogenital disorder-related questions within a specified time frame.

LUDO’s objectives extended beyond token movement, assessing essential skills such as teamwork, time management, resource utilization, and strategic decision-making. The option to seek external resources, limited to five times per team, added an element of strategy. Facilitators evaluated participants’ performance through questionnaires and Likert scales.

The results revealed that LUDO effectively promoted teamwork, communication, leadership, and problem-solving among emergency medicine residents. Resident feedback was overwhelmingly positive, with interest in adopting this format for other educational modules. The gamified approach encouraged engagement and motivation, with immediate feedback ensuring continuous learning.

In conclusion, the incorporation of the LUDO gamified format provides an enjoyable and interactive learning experience for emergency medicine residents. It enhances engagement, fosters teamwork, and facilitates the rapid assimilation of crucial knowledge related to urogenital diseases. This adaptable approach holds promise for improving resident training in various clinical scenarios.

## Background

Urogenital diseases need immediate treatment in the area of emergency care. Ectopic pregnancies, kidney stones, urinary tract infections, testicular torsion, and priapism are only a few of the disorders affecting the urinary and reproductive systems that are included under the umbrella term “urogenital emergencies”. Quick inspection, early diagnosis, and prompt care are crucial for preventing possible mortality and morbidity as well as maintaining patients’ overall health and well-being. Obtaining immediate medical care may be difficult due to the sensitive nature of urogenital illnesses and cultural taboos, which usually involve private and intimate aspects of a person’s health. Implementing cutting-edge gamification-based learning techniques for residents might help them acquire the information they need to get beyond obstacles and problems that can prevent prompt intervention and treatment of urogenital disorders [2]. In order to increase resident engagement, motivation, and information retention, game design approaches are used.

## Method

The Learning Urogenital Diseases in Oddity (LUDO) is a timed, gamification-based exercise with the goal of giving emergency medicine residents an interactive and engaging experience to evaluate their clinical knowledge necessary for the treatment of urogenital disorders. A variation on the well-known game of the same name that has been played for many years in Pakistan is the abbreviation LUDO. By implementing this well-known idea with emergency medicine residents, learning is facilitated and cooperation and collaboration, which are essential in an emergency department, are encouraged. Any learning group may utilize this format to teach courses in an engaging manner because of its customizable simplicity and fun component.

Residents in emergency medicine from all academic years participated in the learning process. Four teams are needed for LUDO, and each team is distinguished by the color it has been given on the game board (Blue, Green, Red, and Yellow).

The roles of the 3 facilitators are as follows:

1. One oversees the handling of the laptop.
2. One indicates how many times (up to a maximum of five) each group has looked for more resources which includes referring to the core textbook or using Internet resources.
3. One serves as an assessor, keeping an eye on their group taking their turn in the game as they roll the dice and move their color piece after answering a question correctly.

### Equipment

**Fig. 1:**
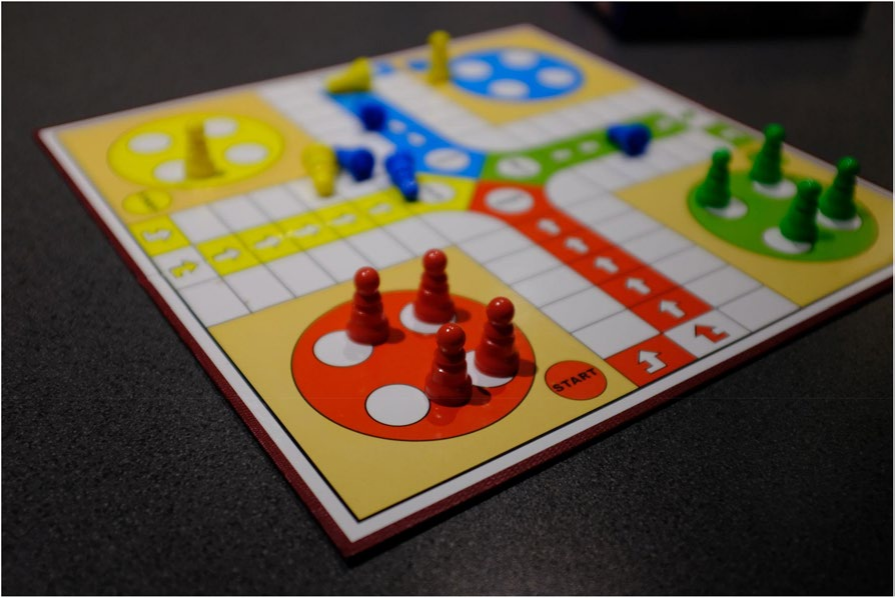
A traditional Ludo board

The exercise makes use of simple, inexpensive, and easily accessible materials, including the following:

- *LUDO board*: A square strategy board (Fig. 1) for 2–4 players, with a pattern in the shape of a cross. In this game, participants race their tokens from start to finish with a single dice roll.
- *LED stopwatch*: An LED stopwatch was mounted next to the projector screen to time the team’s response to each clinical scenario/question. After the facilitator read the question to the team from the laptop, a 1-min timer was started and reset before the following team’s turn.
- *Laptop and projector*: A laptop and projector were used to display PowerPoint slides. On the slides, for example, participants were required to see photos, true/false questions, multiple choice questions, fill-in-the-blank questions, and rapid-fire questions. These questions prompted students to recognize various characteristics of urogenital disorders as well as distinguish photos of genital ulcers that patients may present at urgent care clinics or emergency rooms.
- *Colored hats*: Each person received four different colored hats that matched the game pieces to signify which team they were on.
- Desk bell: If the team member in the “hot seat” desired to answer the question differently than their team’s consensus answer, they could ring the desk bell.

### Summary of the activity

LUDO is an enhanced strategy board game for 2–4 players in which teams compete to advance their four tokens from start to finish using single-die rolls. The activity lasted around 2 h and followed typical LUDO rules with some adjustments.

Before the activity commenced, the activity director explained the program’s rules and regulations, which were also distributed to participants 1 week before the event through email and WhatsApp [2].

**Fig. 2:**
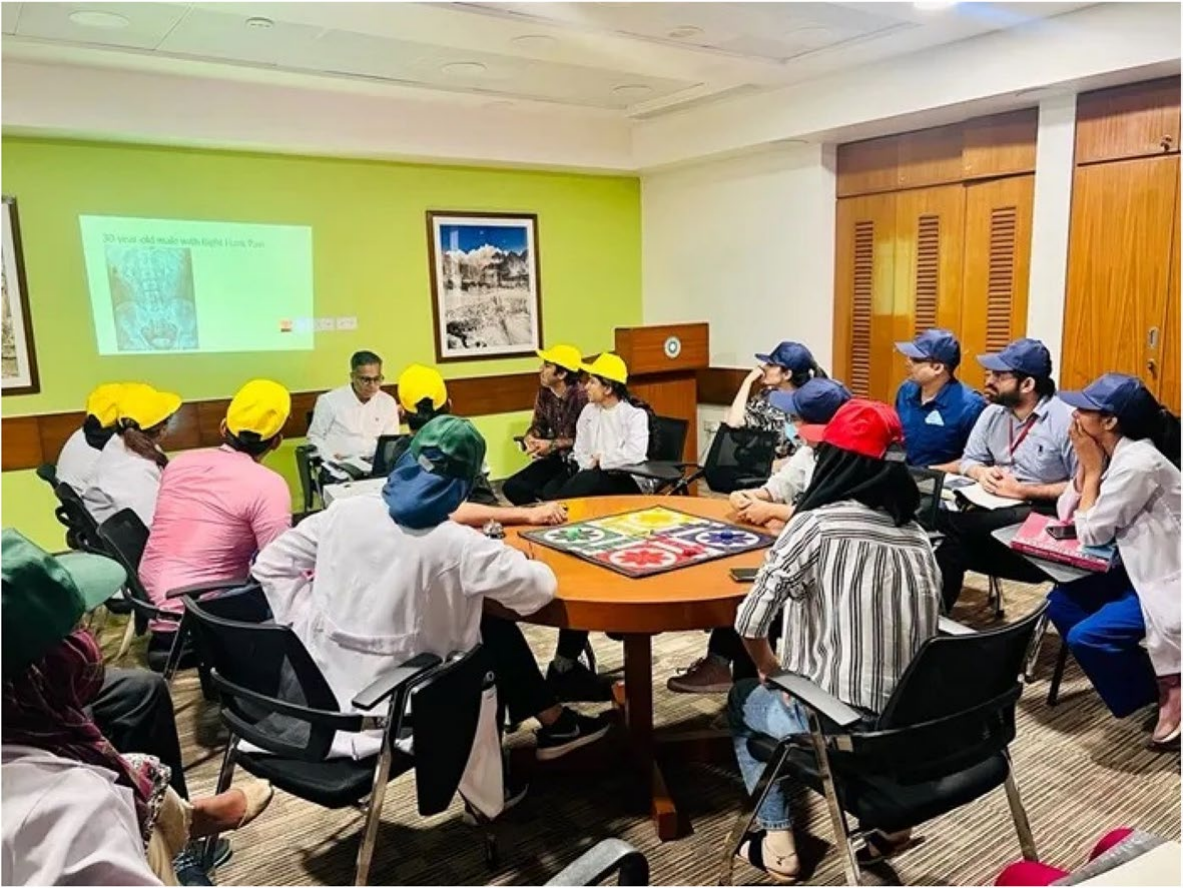
LUDO game with team colors, designated by hats. Team red is listening to the question on data interpretation

In this rendition of LUDO, four team captains assumed the “hot seat” and were in charge of rolling the dice and making the final decision on their team’s replies. To initiate token movement from the home base circle, a roll of six was required, similar to the original game. Following that, teams could only advance if they answered the prepared questions corresponding to each side of the die. Failure to answer a question kept the team in their current place on the board, and the turn was passed on to the next team. After consulting with their team, each team captain had 1 min to respond to a question. An LED clock placed next to the projector screen displayed the remaining time. If a captain wished to provide an alternative answer, differing from the team’s consensus, they could ring the desk bell and present their response (Fig. 2).

**Fig. 3:**
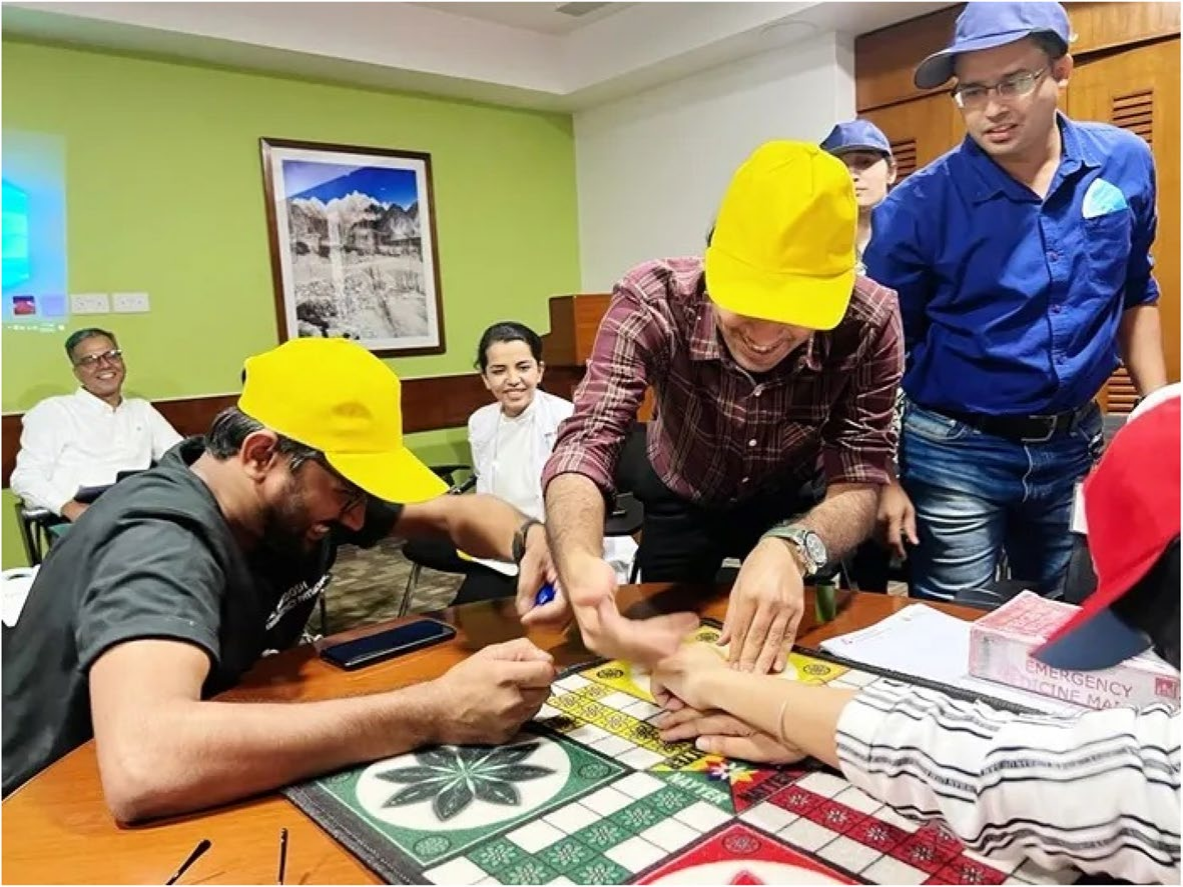
Yellow team captain joyfully removing a red team’s token piece after answering the question correctly

While the final objective of the activity was to get all the tokens home, this was just a part of the evaluation in determining the winner. Other essential skills assessed by the facilitator but not limited to included teamwork, time management, use of external resources (such as books or the Internet), and strategic decision making. For example, deliberately answering a question incorrectly proved to be advantageous at times as it created an opportunity to land on an opponent’s token, causing it to reset back to the home base and start their progress on the board from the beginning (Fig. 3).

Teams were given the option to seek additional resources, such as the core textbook of emergency medicine or using their mobile phones to access resources on the Internet, to aid them in answering questions. However, the use of external resources was limited to 5 times per team and was monitored by a facilitator.

Throughout the activity, the facilitator assessed each team’s performance using a questionnaire with Likert scales to assess various aspects such as knowledge of urogenital diseases, team-captain leadership skills, problem-solving abilities, and communication among team members.

Each team completed an evaluation form at the end of the session to provide feedback to the organizers. Furthermore, at a debriefing session immediately following the activity, facilitators offered timely feedback. The activity director identified and addressed knowledge and skill gaps and offered suggestions on how to utilize the lessons learned in future clinical practice.

In summary, the LUDO-inspired gamification activity provided an engaging and interactive learning experience for EM residents, allowing them to build critical skills and assess their knowledge of urogenital diseases. Participant feedback and debriefing sessions served as valuable tools for improvement and application in real-world medical scenarios [3].

## Results

The activity provided an opportunity for faculty to evaluate critical abilities in Emergency Medicine that go beyond medical expertise, including communication, collaboration, leadership, and problem-solving. EM residents had the chance to practice negotiating team dynamics and working in groups, providing a congenial learning atmosphere for both junior and senior residents.

A 360-feedback strategy was implemented to gather comprehensive feedback. Faculty members used a Likert scale to collect resident feedback on learning outcomes, while residents engaged in mini-interviews to gain educator evaluation after the exercise. A questionnaire was also employed to analyze the effectiveness and learning results of the LUDO gaming activity. This assessment instrument was piloted prior to adoption, and it is now being validated.

The LUDO activity was well received by the EM residents, who expressed interest in repeating and adopting the same format for other educational modules. They found the activity to be a unique learning experience that fostered teamwork and collaboration. The evaluation data was not intended for publication and therefore was not presented in this manuscript.

While the game dynamics can be altered by modifying the complexity of the cases and related questions, these adjustments should align with the intended learning outcomes. It is crucial to preserve the core principles of the game to ensure the essentials of gamification are maintained.

## Conclusion

**Fig. 4:**
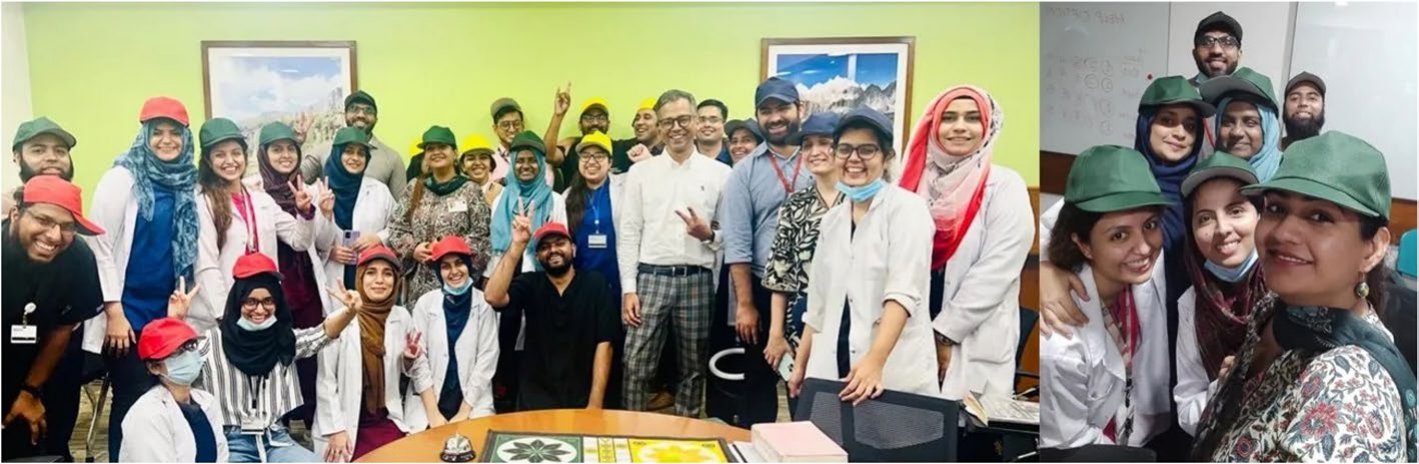
Happy faces at the completion of LUDO (left) and the winning group—team green (right)

The incorporation of gamified format of LUDO provides an enjoyable and interactive learning experience for EM residents, effectively increasing their engagement and motivation levels. The inclusion of timed challenges and active participation within the activity facilitates the swift assimilation and retention of essential facts and clinical practices pertinent to urogenital diseases. Through collaborative effort and strategic planning required to answer clinical scenarios/questions, the activity fosters a sense of teamwork among EM residents. The provision of immediate feedback by the facilitator or the activity director enables continuous learning and clarification of concepts during the activity, enhancing the overall education experience [4]. The flexible nature of the LUDO format allows for easy adaptation to cover other topics of discussion and medical specialties, catering to the needs of different learner groups (Fig. 4).

## Data Availability

Data sharing is not applicable to this article as no datasets were generated or analyzed during the current study.

## Abbreviation

LUDO: Learning Urogenital Diseases in Oddity
